# Vinca alkaloid drugs promote stress-induced translational repression and stress granule formation

**DOI:** 10.18632/oncotarget.8728

**Published:** 2016-04-13

**Authors:** Witold Szaflarski, Marta M. Fay, Nancy Kedersha, Maciej Zabel, Paul Anderson, Pavel Ivanov

**Affiliations:** ^1^ Division of Rheumatology, Immunology, and Allergy, Brigham and Women's Hospital, Boston, MA, USA; ^2^ Department of Medicine, Harvard Medical School, Boston, MA, USA; ^3^ Department of Histology and Embryology, Poznan University of Medical Sciences, Poznań, Poland; ^4^ The Broad Institute of Harvard and M.I.T., Cambridge, MA, USA

**Keywords:** chemotherapy, stress granules, translation initiation, stress response, cancer

## Abstract

Resistance to chemotherapy drugs is a serious therapeutic problem and its underlying molecular mechanisms are complex. Stress granules (SGs), cytoplasmic ribonucleoprotein complexes assembled in cells exposed to stress, are implicated in various aspects of cancer cell metabolism and survival. SGs promote the survival of stressed cells by reprogramming gene expression and inhibiting pro-apoptotic signaling cascades. We show that the vinca alkaloid (VA) class of anti-neoplastic agents potently activates a SG-mediated stress response program. VAs inhibit translation initiation by simultaneous activation of eIF4E-BP1 and phosphorylation of eIF2α, causing polysome disassembly and SG assembly. VA-induced SGs contain canonical SG components but lack specific signaling molecules. Blocking VA-induced SG assembly by inactivating eIF4EBP1 or inhibiting eIF2α phosphorylation decreases cancer cell viability and promotes apoptosis. Our data describe previously unappreciated effects of VAs on cellular RNA metabolism and illuminate the roles of SGs in cancer cell survival.

## INTRODUCTION

Tumor cells reside in inhospitable environments that select for cells that acquire adaptive mechanisms that promote their growth and survival. One of the most important stress-activated adaptive mechanisms is the ability to reprogram protein translation in a way that conserves anabolic energy for the repair of stress-induced damage. Inhibition of cap-dependent translation initiation dampens the production of most cellular proteins, while preserving the production of proteins encoded by transcripts possessing internal ribosome initiation sites or 5′-upstream open reading frames which commonly encode proteins that enhance the survival of cells exposed to adverse conditions (discussed in [1–3]).

At the molecular level, two main pathways control mRNA translation initiation. The first is the phosphorylation of initiation factor 2 alpha (eIF2α), a component of the ternary complex that delivers initiator tRNA to translation-competent pre-initiation complexes at the 5′ ends of capped mRNA. Phosphorylation of eIF2α at serine 51 (S51) by one of four stress-activated eIF2α kinases (PKR, PERK, GCN2 and HRI) prevents ternary complex assembly and thus inhibits translation initiation. The second control point regulates the assembly of the eIF4F (i.e. eIF4E:eIF4G:eIF4A) complex, controlled by the PI3K-mTOR (mammalian target of rapamycin) kinase cascade. Stress-induced inactivation of mTOR leads to the activation of its down-stream target, eIF4E-binding protein (e.g. eIF4E-BP1 (4E-BP1)). Activated 4E-BP1 prevents the assembly of eIF4F leading to inhibition of translation initiation. Although both pathways play complementary roles in the control of translation, they also allow targeted translational control of specific mRNA subsets [1–3].

Transcripts subject to stress-induced translational arrest are often actively compartmentalized into discrete cytoplasmic foci known as stress granules (SGs) [4, 5]. SGs are large ribonucleoprotein (RNP) assemblies composed primarily of stalled translation initiation complexes and a plethora of RNA-binding proteins and signaling proteins involved in various aspects of cellular metabolism. SGs are not passive mRNA storage sites; they are dynamic entities that determine the fate of specific transcripts shuttling through them, and additionally modulate various signaling cascades to determine whether stressed cells will live or die. Dysregulation of SG dynamics is implicated in the pathogenesis of a number of human diseases including cancer [6].

In most cases, the goal of cancer chemotherapy is to maximally damage and ultimately kill cancer cells. Recent data suggests that cancer cells use stress-adaptive responses targeting the translational machinery to adapt/survive chemotherapy treatments [7, 8]. We have made the surprising discovery that vinca alkaloids (VA), cytotoxic anti-mitotic drugs targeting microtubules[9], are potent inducers of translational repression and SG formation, despite the lack of previous reports implicating VA involvement in RNA metabolism. VA-induced translational repression affects both mTOR/4E-BP and phospho-eIF2α cascades to target translation initiation and promote SG formation. Interference with phospho-eIF2α and 4E-BP functions significantly affects cancer cell viability and apoptosis, making these pathways potential targets for cancer therapy. Moreover, cancer cells that are genetically modified and unable to assemble SGs are more vulnerable to VA-induced cell death. These results reveal an important role for translational control and SGs in drug resistance of cancer cells.

## RESULTS

### Vinca alkaloids are potent inducers of stress granules

**Figure 1:**
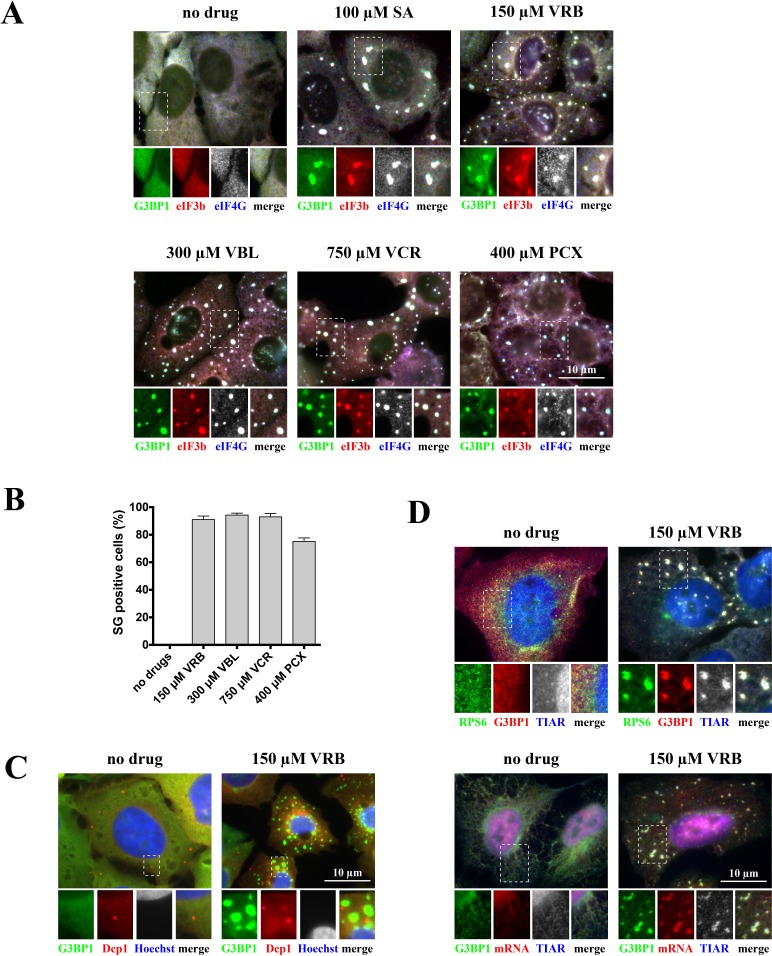
Vinca alcaloids induce formation of SGs **A.** U2OS cells were stressed with sodium arsenite (SA, 100 μM), vinorelbine (VRB, 150 μM), vinblastine (VBL, 300 μM), vincristine (VCR, 750 μM) and paclitaxel (PCX, 400 μM) for 1 hour. Unstressed U2OS cells (no drug) were used as control. After treatment, cells were stained for SG markers G3BP1 (green), eIF4G (blue, shown as gray), eIF3b (red) and scored. Boxed region is shown enlarged with colors separated below each image; merged signals shown as gray. Size bar represents 10 μm. **B.** Quantification of SG-positive U2OS cells (as in Figure 1A). Data were analyzed using the unpaired Student's *t*-test, *N* = 3. **C.** Vinorelbine does not affect P-bodies. U2OS WT cells were stressed with vinorelbine (VRB, 150 μM) or left untreated (no drug) for 1 hour and then stained with P-body marker Dcp1 (red) or SG marker G3BP1 (green). Nuclei are visualized with Hoechst staining (blue). Boxed region is shown enlarged with colors separated below each image. Size bar represents 10 μm. **D.** VRB-induced SGs contain mRNAs and 40S ribosomal subunits. U2OS WT cells stably expressing ribosomal protein S6 (RPS6) fused to GFP (GFP-RPS6) were stressed with vinorelbine (VRB, 150 μM) or left untreated (no drug) for 1 hour. Upper panel: Cells were visualized for GFP-RPS6 (green) or SG markers G3BP1 (red) and TIAR (blue, shown as gray). Lower panel: Cells were stained with SG markers G3BP1 (green) and TIAR (blue, shown as gray). *In situ* hybridization with oligo-dT_40_ probe against polyadenylated mRNAs (red) was done as described. Boxed region is shown enlarged with colors separated below each image. Size bar represents 10 μm.

To determine whether certain chemotherapy drugs induce SG formation, we performed an unbiased screening by challenging osteosarcoma U2OS cells with a library of FDA-approved chemotherapy drugs (~25 compounds) using variable drug concentrations (data not shown). SG formation was assessed by immunofluorescence using antibodies against canonical SG components (such as G3BP1, eIF4G and eIF3b, Figure 1). We identified chemotherapy drugs targeting the microtubule network as potent inducers of SGs (Figure 1A and 1B). These drugs include both anti-mitotic microtubule destabilizing members of the vinca alkaloid (VA) family, including vinorelbine (VRB), vinblastine (VBL) and vincristine (VCR), and the microtubule-stabilizing taxane family member paclitaxel (PCX). The VA concentrations that induce SG formation are within the range of the drugs' IC_50_ values in U2OS cells (Figure S1). We observed that VA-induced SGs are generally smaller than sodium arsenite (SA)-induced SGs at early treatment times (~1 hour), but become progressively larger with longer treatment (~4 hours) (Figure S2A). As both VAs and taxane drugs target microtubules, we also determined the effects of different doses of these drugs on microtubule networks. While VRB acts on tubulin to inhibit microtubule formation (i.e., destabilizes microtubule networks), PCX prevents the breakdown of microtubules (i.e., stabilizes and prevents their disassembly). Drug treatments that efficiently collapse (Figure S2B: left panel, 150μM of VRB) or stabilize (Figure S2B: right panel, 400μM of PCX) microtubule networks promote SG formation. Under VRB treatment, tubulin aggregates into tubulin-positive inclusions that do not contain SG markers, although SGs themselves are often found in the vicinity of these aggregates. Similarly, tubulin is not found in SGs in cells treated with PCX (Figure S2B).

In the subsequent experiments aimed at determining the composition and function of VA-induced SGs, we chose to focus on VRB, which produces a robust, dose- and time- dependent SG response (Figure S3A-B). In addition to U2OS osteosarcoma cells, VRB potently induces SGs in other cancer cell lines including SiHa (cervix), MCF7 (breast) and A549 (lung) (Figure S3C) using similar concentrations.

### Composition of VA-induced SGs

The subcellular distribution and morphology of VA-induced SGs resembles that of SA-induced SGs (Figures 1, S2 and S4) [10]. VRB-induced SGs contain core SG components including poly(A)-containing mRNAs (assessed by fluorescence *in situ* hybridization (FISH) using oligo-dT (Figure 1D, lower panel)), small ribosomal subunits (assessed by detection of the ribosomal protein RPS6 (Figure 1D, upper panel)) and the classical SG marker TIAR (Figure 1C, upper/lower panels). Further analysis shows that VRB-induced SGs recruit poly(A)-binding protein (PABP), initiation factors eIF4E and eIF4A, CAPRIN1 and USP10 (G3BP-binding partners), translation modulators TIA1, HuR, FMR1, FXR1 and the microtubule-associated RNA-binding protein STAU1 (Figure S4). VRB-induced SGs do not contain the P-body marker Dcp1, although they are often found in physical proximity to P-bodies (Figure 1C).

**Figure 2:**
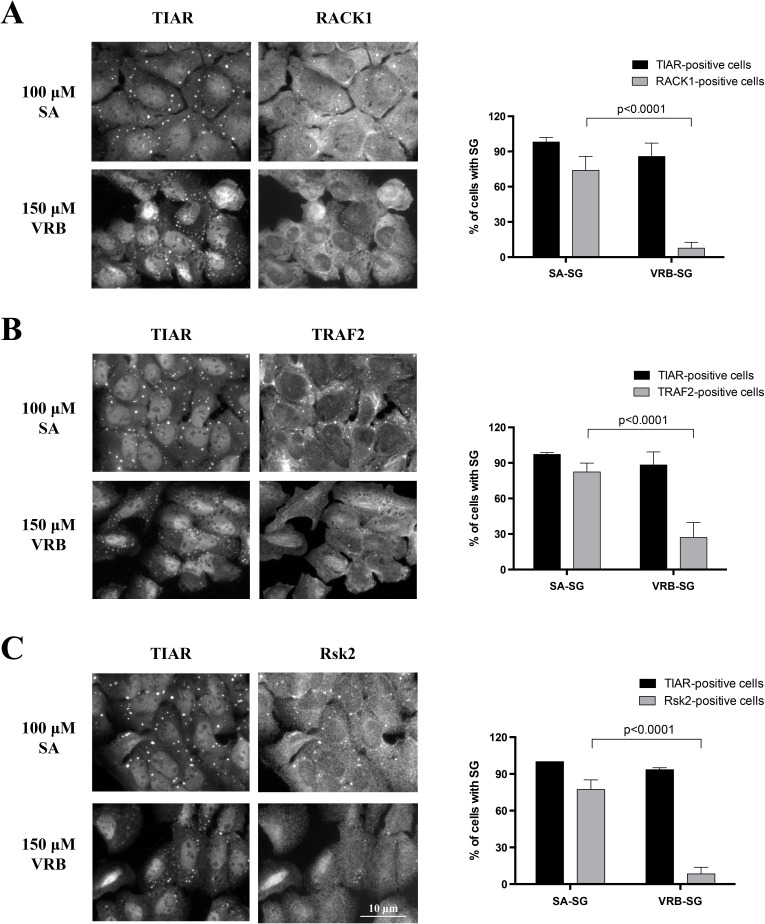
VRB-induced SGs lack specific signaling molecules **A.**-**C.** U2OS cells were stressed with sodium arsenite (SA, 100 μM) or vinorelbine (VRB, 150 μM) for 1 hour. Signaling molecules RACK1 (A), TRAF2 (B) and Rsk2 (C) were probed for SG localization by co-immunostatining with SG marker TIAR. Percentage of TIAR-positive and RACK1- (A), TRAF2- (B) and Rsk2- (C) positive cells is shown on the right panel. Data were analyzed using the unpaired Student's t-test, N = 3. p-values are shown.

These data reveal that VRB-induced SGs contain the major “canonical” components of SGs. Recent data suggest that although SGs show little variation in the recruitment of core components, they can differ in their recruitment of select signaling and apoptosis-related molecules (reviewed in [6]). As shown in Figure 2, localization of RACK1 [11], TRAF2[12] and RSK2[13] into SA-induced SGs is more robust than that observed in VRB-induced SGs, suggesting that these drugs may use different mechanisms to assemble SGs and modulate cell survival.

**Figure 3:**
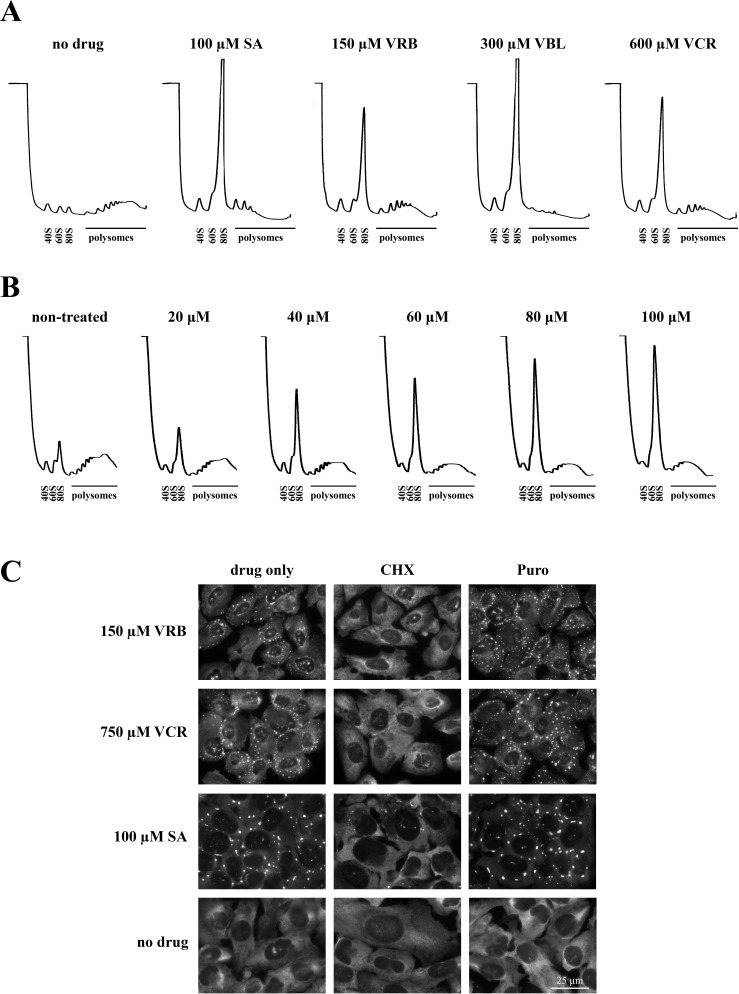
VRB induces polysome disassembly and formation of *bona fide* SGs **A.** Polysome profiles obtained from U2OS cells treated with sodium arsenite (SA, 100 μM), vinorelbine (VRB, 150 μM), vinblastine (VBL, 300 μM) and vincristine (VCR, 750 μM) for 1 hour. Unstressed U2OS cells (no drug) were used as control. **B.** Dose-dependent VRB-induced polysome disassembly. Polysome profiles were obatained from U2OS cells treated with indicated concentrations of VRB for 1 hour. **C.** VRB-induced RNA granules are *bona fide* SGs. VRB- and VCR-induced SGs are disassembled by cycloheximide (CHX) and promoted by puromycin (Puro). SGs are visualized by G3BP1 staining (green, shown as gray). Nuclei are stained with Hoechst (blue, shown as gray). Size bar represents 25 μm.

SGs are dynamic ribonucleoprotein structures [14] that exist in equilibrium with polysomes. Treatment with SA effectively collapses polysomes, resulting in increased levels of 80S monosomes and 40S/60S ribosomal subunits as shown in polysome profiles obtained using sucrose gradient centrifugation (Figure 3A, 100μM SA). Treatment with VA drugs (150μM VRB, 300μM VBL and 600μM VCR) also effectively disassembles polysomes (Figure 3A) suggesting that VA drugs directly or indirectly influence cellular translation. VRB-induced polysome disassembly is dose-dependent with effective concentrations as low as 20 μM (Figure 3B). At the molecular level, pharmacological manipulations that affect polysome dynamics also alter SG assembly and disassembly. Cycloheximide (CHX), a drug that arrests translation elongation and stabilizes polysomes, promotes the disassembly of both SA-induced and VRB-induced SGs (Figure 3C, CHX [15]). In contrast, puromycin (Puro, a translation inhibitor that collapses polysomes by premature termination [15]) promotes the formation of both SA- and VRB-induced SGs (Figure 3C, Puro). Collectively, these data indicate that VA-induced SGs are the *bona fide* SGs (Figures 1 and 3).

### Vinca alkaloids promote SG formation in a phospho-eIF2α dependent manner

**Figure 4:**
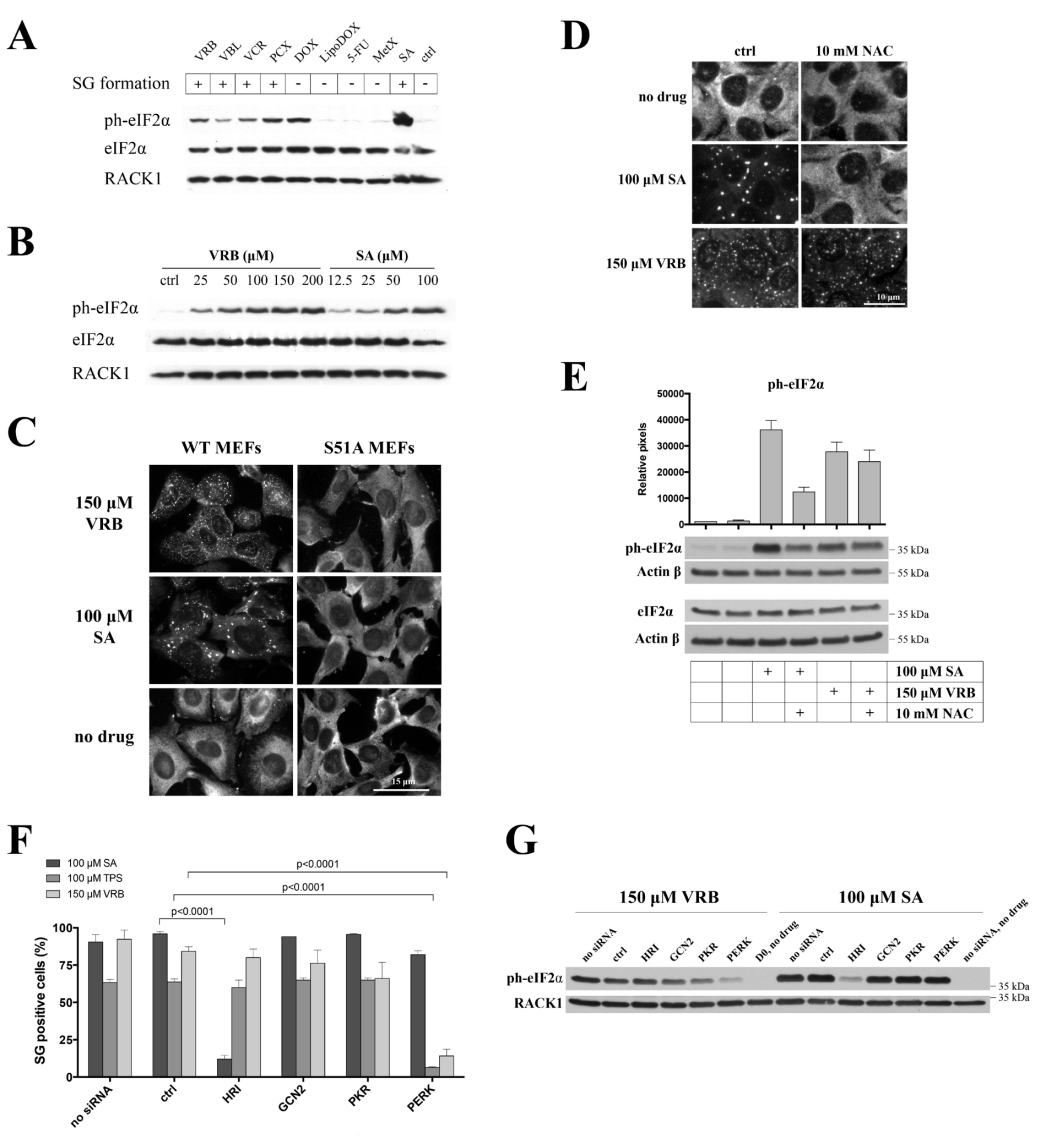
VRB promotes eIF2α phosphorylation *via* activation of PERK kinase **A.** U2OS cells were treated with 100 μM VRB, VBL, VCR, PCX, doxorubicyn (DOX) and its liposome-conjugated form (LipoDOX), 5-fluorouracil (5-FU), methotrexate (MetX) or SA for 1 hour. Untreated cells were used as control (ctrl). Levels of ph-eIF2α were determined by western blotting using ph-eIF2α-specific antibodies. RACK1 and total eIF2α were used as controls for loading. SG formation is indicated in upper boxes. **B.** Dose-dependent phosphorylation of eIF2α by VRB. Western blotting for ph-eIF2α is done as above. SA treatment is used as control. **C.** Wild type MEFs (WT MEFs) or MEFs bearing S51A mutant eIF2α (S51A MEFs) were treated with VRB or SA for 1 hour, and stained with SG marker eIF3b. Untreated cells (no drug) served as control. Nuclei were revealed with Hoechst staining (nuclei). Size bar represents 15 μm. **D.** U2OS cells were treated with SA or VRB in the absence (ctrl) or presence of 10mM *N*-acetylcysteine (NAC) for 1 hour. SGs were revealed by staining with G3BP1. Size bar represents 10 μm. **E.** Quantification of ph-eIF2α levels by western blotting and densitometry (ImageJ) from three independent experiments (as in Figure 4D). Actin is used as loading control. **F.** Quantification of SGs in non-treated U2OS cells (no siRNA) or treated with control - (ctrl), HRI-, GCN2-, PKR- and PERK-specific siRNAs followed by SA, thapsigargin (TPS) or VRB treatments (100 μM, 1hour). Statistical data were analyzed using the unpaired Student's *t*-test (p-values are shown, *N* = 3). Actual levels of HRI-, GCN2-, PKR- and PERK can be seen in S5A-B. **G.** Non-treated (no siRNA), control siRNA-treated (ctrl) or HRI-,GCN2, PKR- or PERK-depleted U2OS cells were treated with 100 μM of VRB or SA for 1 hour. Whole cell lysates were subjected to western blotting using ph-eIF2α-specific antibodies. RACK1 was used as loading control.

Mechanistically, SGs are assembled in response to inhibition of translation initiation [16]. VAs disassemble polysomes in a manner similar to that of SA (Figure 3A-3B), which triggers phosphorylation of eIF2α to inhibit translation initiation. Indeed, VAs and PCX trigger phosphorylation of eIF2α (Figure 4A, lanes VRB, VBL, VCR, PCX compared to control-treated (ctrl) or methotrexate (MetX)), albeit somewhat less robustly than SA (Figure 4A, SA) in U2OS cells. Generally, the ability of VAs to induce the phosphorylation of eIF2α correlates with their ability to promote SGs (Figure 4A, “SGs”). We have noticed, however, that other chemotherapy drugs (tested in our initial screening) do not show a direct correlation between SG formation and eIF2α phosphorylation. For example, doxorubicin (DOX), but not its liposome-conjugated form (LipoDOX), efficiently triggers phospho-eIF2α while neither form of the drug promotes SG formation (Figure 4A and data not shown). Similarly, Fluorouracil (5-FU) is reported to both trigger SG assembly and increase phosphorylation of eIF2α following prolonged treatment [17] but not under short time treatment in our system (Figure 4A). VRB triggers eIF2α phosphorylation in a dose-dependent manner (Figure 4B).

Some stresses (e.g., SA [15]) but not others (e.g., hydrogen peroxide (H_2_O_2_) [18] or selenite [13]) strictly require eIF2α phosphorylation in order to promote SG formation. To determine whether VA-induced SG assembly is phospho-eIF2α dependent, we used a mouse embryonic fibroblast (MEF) line in which wild type (WT) eIF2α (WT MEFs) is replaced with a non-phophorylatable knock-in mutation Ser51Ala (S51A MEFs) [19]. As shown in Figure 4C, both SA and VRB promote SG formation in WT MEFs (as well as in U2OS cells) but not in S51A MEFs, indicating that eIF2α phosphorylation is required for VRB-induced SG assembly.

Phosphorylation of eIF2α results from activation of one or more stress-sensing serine/threonine kinases [20], including GCN2 (activated by amino acid deprivation [21]), HRI (monitors oxidative stress/ROS levels [22]), PERK (senses endoplasmic reticulum (ER) stress [23, 24]) and PKR (activated by double-stranded RNA during viral infections, UV exposure and heat shock [25]). In some cells (e.g., endothelial cells), VRB causes the accumulation of reactive oxidative species (ROS) that promote oxidative stress [26], which may then cause SG formation by HRI activation. To determine whether ROS contributes to VRB-induced SG assembly, we treated U2OS cells with *N*-acetylcysteine (NAC), a common ROS scavenger and antioxidant [27], together with VRB. As seen in Figure 4D, NAC efficiently inhibits SA-induced but not VRB-induced formation of SGs. In agreement with these results, VRB-induced phosphorylation of eIF2α is not affected by NAC treatment. In contrast, SA-induced phosphorylation of eIF2α is modestly inhibited by NAC treatment (Figure 4E).

To identify the eIF2α kinase activated by VRB, we used siRNA to deplete GCN2, PKR, HRI and PERK kinases in U2OS cells, and then subjected kinase-depleted cell lines to VRB treatment followed by SG quantifications (Figure 4F and Figure S5). Depletion of PERK significantly inhibits VRB-induced (Figure 4F and Figure S5) and thapsigragin (TPS)-induced SG formation (TPS triggers PERK/phospho-eIF2α/SGs and used as a positive control). Similarly, direct quantification of phospho-eIF2α levels in PERK-depleted U2OS cells suggests that PERK is activated by VRB treatment (Figures 4G, SA treatments is used as a control for HRI activation). Taken together, these results identify PERK as the VRB-activated eIF2α kinase.

### Vinca alkaloids inhibit mTOR and activate eIF4E-BP1 to disrupt the eIF4F complex

**Figure 5:**
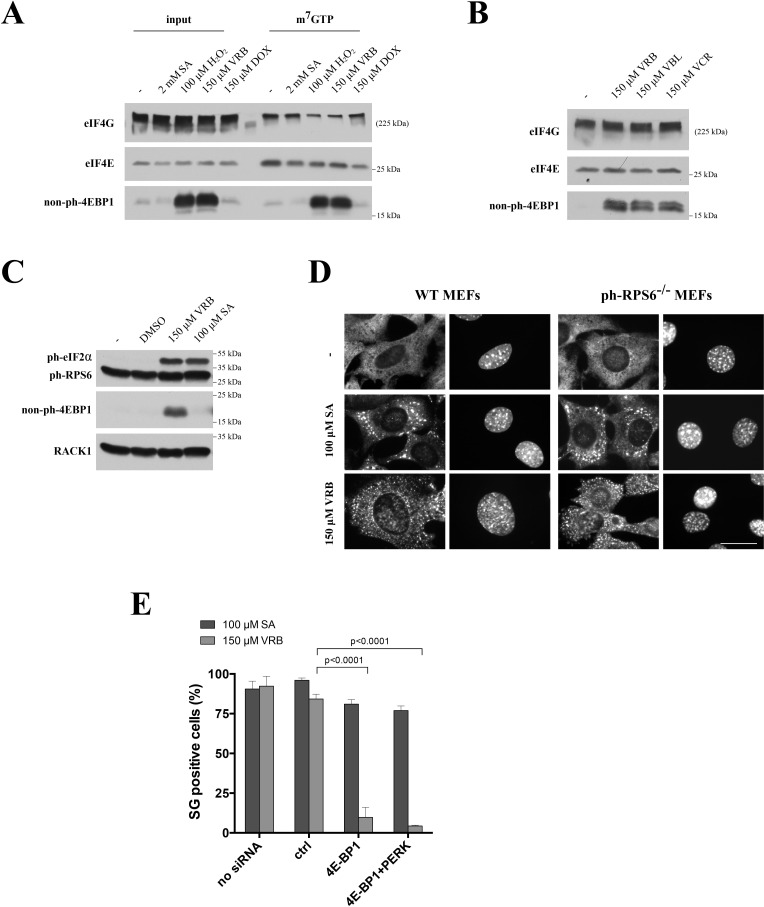
VRB modulates mTOR/4E-BP1 to promote eIF4F complex remodeling and SG formation **A.** VRB disrupts eIF4F complex formation and enhances eIF4E/4E-BP1 interactions. U2OS cells without (−) or with VRB (150 μM), SA (100 μM), DOX (150 μM) or hydrogen peroxide (H_2_O_2_, 1mM) treatment (1hour) were lysed and subjected to m^7^GTP-sepaharose pull down to isolate cap-bound complexes. Both the input (input) and precipitated (m7GTP) fractions were subjected to western blotting and probed against eIF4G, eIF4E and non-phosphorylated 4E-BP1 (non-ph-4EBP1). **B.** The same as in 5A, except that U2OS cells were treated with indicated VAs and levels of non-phosphorylated 4E-BP1 (non-ph-4EBP1) were compared to control treatment (−). Levels of eIF4G and eIF4E were served as controls. **C.** VRB treatment does not affect ph-RPS6 status. Whole cell lysates from VRB-, SA-, DMSO-treated and untreated (−) U2OS cells were subjected to western blotting using ph-eIF2α-, ph-RPS6 and non-ph-4E-BP1-specific antibodies. RACK1 was used as loading control. **D.** VRB-induced SG formation in MEFs carrying non-phosphorylatable variant of RPS6 (ph-RPS6^−/−^). WT and ph-RPS6^−/−^ MEFs were subjected to no stress (−), 100 μM SA and 150 μM VRB and stained with G3BP1 and Hoechst (nuclei). Size bar represents 10 μm. **E.** Depletion of 4E-BP1 inhibits VRB-induced SG formation. WT (no siRNA), control siRNA- (ctrl), 4E-BP1- and 4E-BP1/PERK-treated U2OS cells subjected to 100 μM SA and 150 μM VRB, stained with G3BP1 and % of SG-positive cells was determined. Standard statistics was applied (unpaired Student's *t*-test (p-values are shown, *N* = 3)).

VAs induce PERK-mediated eIF2α phosphorylation that contributes to SG assembly. In addition to phospho-eIF2α, some chemotherapy drugs (such as selenite [13]) and oxidative agents (H_2_O_2_ [18]) also target the eIF4F complex to promote SG formation. Both selenite and H_2_O_2_ induce 4E-BP1:eIF4E interactions that sequester eIF4E away from the eIF4F complex [13, 18]. To determine whether disruption of the eIF4F complex contributes to VRB-induced SG formation, we examined the integrity of the eIF4F complex in SA-, H_2_O_2_-, DOX- and VRB- treated U2OS cells (note that all these treatments result in phosphorylation of eIF2α). As shown in Figure 5A, none of the treatments alters the expression of eIF4E, eIF4A or eIF4G proteins (input). Importantly, VRB treatment promotes dephosphorylation of 4E-BP1 in a manner similar to H_2_O_2_ (and as reported in [18]). Pulling down eIF4E-containing complexes by m^7^GTP Sepharose (m^7^GTP) revealed that neither treatment affects eIF4E:m^7^GTP interactions that mimic the binding of eIF4E to the 5′-cap structures of mRNAs. At the same time, H_2_O_2_ and VRB selectively increase eIF4E:4E-BP1 interactions, leading to the competitive displacement of eIF4G and eIF4A from m^7^GTP-bound eIF4E; neither SA nor DOX disrupt the eIF4F complex (Figure 5A). Further analysis suggests that the ability to trigger dephosphorylation of 4E-BP1 is a common activity of VA family members (Figure 5B). Together these data indicate that VRB disrupts eIF4F complex formation by promoting dephosphorylation of 4E-BP1.

VRB-induced translational repression mediated by 4EBP1:eIF4E interactions strongly implicates mTOR kinase, the upstream regulator of 4EBP1, in this process. As mTOR also signals to translation by activating the ribosomal protein S6 kinase (p70S6K) to phosphorylate the ribosomal protein S6 (RPS6) [28], we determined the effects of VRB on RPS6 phosphorylation (phospho-RPS6). The levels of phospho-RPS6 are unchanged following treatment with VRB or SA, in contrast to the levels of phospho-eIF2α (Figure 5C). In addition, VRB treatment promotes formation of SGs in MEFs carrying a non-phosphorylatable variant of RPS6 (phospho-RPS6^−/−^) (Figure 5D) [29]. These data support a specific inhibition of the mTOR/4EBP1 but not the mTOR/p70S6K/p-RPS6 axis by VRB. Finally, siRNA-mediated depletion of 4E-BP1 inhibits VRB-induced SG formation to comparable levels as does PERK depletion (note, that 4E-BP1 depletion does not trigger eIF2α phosphorylation, Figure S5C). Simultaneous depletion of PERK and 4E-BP1 inhibits SG assembly by VRB stronger than single depletions (Figure 5E). Thus, both PERK and 4E-BP1 contribute to VRB-induced SG formation, although PERK-mediated contribution seems to play the major role.

### Role of PERK/4E-BP1/stress granules axis on vinorelbine-induced toxicity

**Figure 6:**
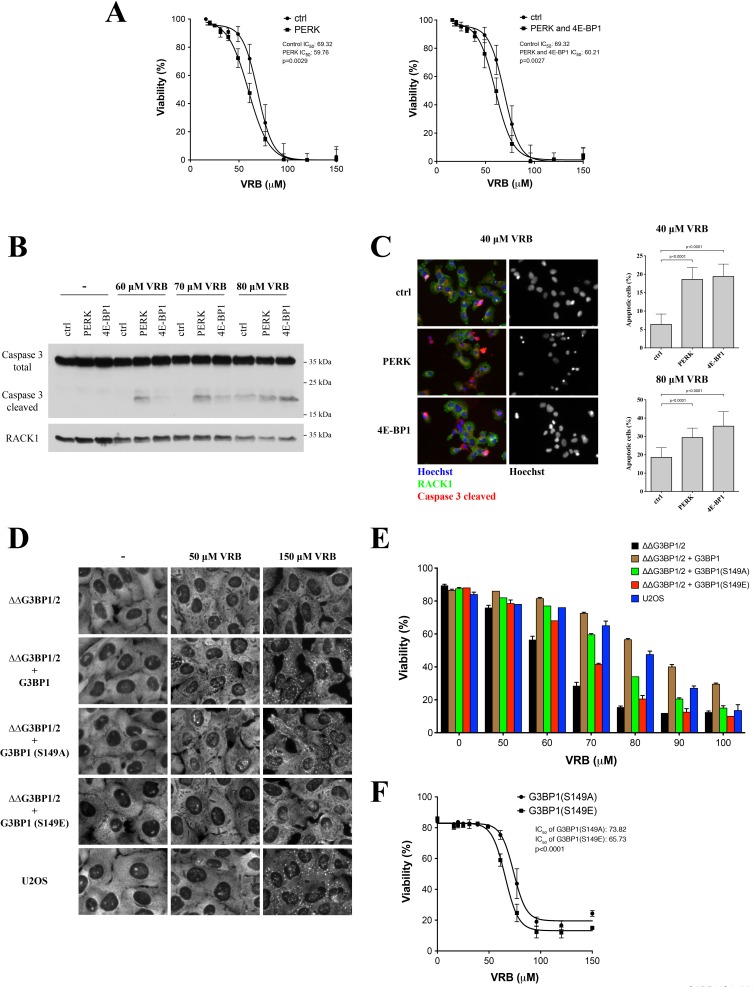
Effects of SGs and 4E-BP1/PERK knockdown on the viability of VRB-treated cells **A.** Viability of control siRNA-, PERK- and PERK/4E-BP1-depleted cells in the presence of various VRB concentrations (24 hours). Viability was determined by measuring the degree of cell death. The half maximal inhibitory concentrations (IC_50_) of VRB are shown. **B.** U2OS cells depleted of PERK or 4E-BP1 were subjected to 60, 70, 80μM of VRB for 1 hour. Control siRNA-treated cells (ctrl) were used as control. Whole cell lysates from VRB and untreated (−) U2OS cells (ctrl, PERK and 4E-BP1) were subjected to Western Blotting using Caspase 3- (inactive) and cleaved form of Caspase 3 (active)-specific antibodies. RACK1 was used as loading control. **C.** Percentage of cells undergoing apoptosis was determined by immunofluorescence using cleaved Caspase 3-specific antibodies (red) on populations of control siRNA-treated, PERK- and 4E-BP1-depleted cells subjected to 40 or 80 μM VRB. Representative IF images are shown. Standard statistics were applied (unpaired Student's *t*-test (p-values are shown, *N* = 3)). **D.** VRB-induced SG formation in SG-competent (U2OS, ΔΔG3BP1/2+G3BP1, ΔΔG3BP1/2+ S149A) and SG-incompetent (ΔΔG3BP1/2, ΔΔG3BP1/2+S149E) cells. Representative images with no treatment (−), 50 μM and 150 μM VRB are shown. eIF4G was used as a SG marker. **E.** Viability of SG-competent and incompetent U2OS cells (Figure 6D) treated with indicated VRB concentrations (0-100 μM, 24h). **F.** The half maximal inhibitory concentrations (IC50) of VRB and viability of ΔΔG3BP1/2 cells reconstituted with SG-competent S149A and SG-incompetent S149E mutants (24 h). Standard statistics was applied (unpaired Student's *t*-test (p-values are shown, *N* = 3)).

To examine the physiological roles of PERK and 4E-BP1 in the VRB-induced stress response, we compared the cytotoxicity of various VRB concentrations of PERK- and PERK/4E-BP1-depleted U2OS cells. As shown in Figure 6A, the toxicity of VRB in PERK- and PERK/4E-BP1-depleted U2OS cells is greater than that observed in control siRNA-treated cells. Note, that double depletion (PERK/4E-BP1) has a very similar response to VRB treatment to the single PERK knockdown, in agreement with major role of eIF2α phosphorylation in VA-induced SG formation (Figure 4C). VRB-induced cell death is primarily apoptotic as assessed by the appearance of cleaved Caspase 3 in western blots of cell lysates (Figure 6B) and immunostaining (Figure 6C). Importantly, in U2OS cells depleted of PERK and/or 4E-BP1, activation of apoptosis is more evident at lower concentrations of VRB than in control siRNA-treated cells (Figure 6B-6C).

PERK and 4E-BP1 are required for VRB-mediated SG formation (Figures 4F and 5E) but their depletion may affect other pathways and have pleiotropic effects. In order to ask whether SGs directly contribute to cell resistance/survival in response to VRB, we used CRISPR/CAS9-modified U2OS cells lacking both G3BP1 and G3BP2 (ΔΔG3BP1/2), related proteins that are absolutely required for all phospho-eIF2α-dependent and some phospho-eIF2α independent SG formation [30]. VRB fails to induce SGs in ΔΔG3BP1/2 cells but successfully induce SGs in ΔΔG3BP1/2 cells reconstituted with WT G3BP1 (ΔΔG3BP1/2+G3BP1) (Figure 6D). The SG-promoting ability of G3BP is inhibited by phosphorylation at Serine 149; G3BP1-S149A, a non-phosphorylatable variant rescues SGs while the phosphomimetic variant G3BP1-S149E fails to rescue SG formation in ΔΔG3BP1/2 cells [30]. VRB promotes SG formation in ΔΔG3BP1/2 cells reconstituted with G3BP1-S149A but not with G3BP1-S149E (Figure 6D). Importantly, VRB resistance correlates with SG formation; cells expressing SG-promoting G3BP1 WT or S149A are more resistant to VRB-induced cell death than are cells expressing the SG-deficient G3BP1 S149E variant (Figure 6E and 6F). Together with the PERK/4E-BP1 depletion data, these results suggest that SGs in promote cell survival in response to VRB treatment.

## DISCUSSION

While SG formation is often associated with cell survival and inhibition of apoptosis, their formation in response to chemotherapy drugs can contribute to both cancer cell resistance and sensitivity [6]. To date, several chemotherapeutic drugs have been reported to induce SGs. These SG-inducing drugs are chemically diverse and target various cellular processes. With the exception of sodium selenite which induces pro-apoptotic SGs (non-canonical SGs lacking eIF3), all other known drugs promote cytoprotective SGs. The assembly of SGs by chemotherapy drugs requires the phosphorylation of eIF2α, inactivation of mTOR signaling or both. Proteasome inhibitors Bortezomib (PS-341/Velcade) [31] and MG132 [32], the antimetabolites 5-Fluorouracil and 5-azacytidine [33], and the kinase inhibitor Sorafenib [34] induce phospho-eIF2α to trigger SG formation, while sodium selenite additionally acts *via* the mTOR/4E-BP1 axis [13].

We now identify VAs as potent inducers of *bona fide* SGs. The ability of VAs to promote SG formation is unexpected as another microtubule-disrupting agent, nocodazole, had been reported to inhibit SG formation, although reports are conflicting ([35–39], reviewed in [40]). The main difference between these and our studies is in the doses and treatment regimes of microtubule-modulating drugs as well as in the use of the different cell lines. Many studies monitored specific effect of microtubule-modulating drugs on sodium arsenite-induced SG formation, thus further influencing cellular metabolism, while our study monitors SGs induced by the VA class of microtubule-modullating drugs. Moreover, nocodazole or colchicine treatments do not directly affect phospho-eIF2α and/or mTOR signaling pathways. Our data suggest that lower doses of VA drugs promote destabilization of microtubule networks, promote translation repression but do not induce formation of microscopically visible SGs. Higher concentrations or prolonged treatment potently promote translation repression and also induce microscopically visible SGs. We speculate that higher doses may promote secondary aggregation/coalescence of SGs as a result of robust polysome disassembly caused by the collapse of the microtubule network. In agreement large VRB-induced SGs are often found in the vicinity of drug-induced tubulin aggregates (Figure S2B), although these structures do not colocalize. The ability of VAs to induce SGs does not rely solely on their ability to interfere with microtubule dynamics, as both lower and higher VA concentrations are sufficient to disrupt microtubules. Rather, their ability to promote SGs is dependent on translation repression, a previously unappreciated aspect of VA's effects on cellular metabolism.

Our results identify phospho-eIF2α and mTOR signaling cascades as mediators of VA-induced translational repression. VRB-induced eIF2α phosphorylation is evident at concentrations as low as 25 μM (Figure 4B), which correlates with VRB-induced polysome disassembly (Figure 3B). It should be noted that low concentrations of SA can trigger phosphorylation of eIF2α (e.g. at 12.5-25 μM, Figure 4B) but not the formation of detectable (microscopically visible) SGs, similar to results with VRB. In contrast to SA, which activates HRI, VRB activates PERK, as siRNA-mediated depletion of PERK down-regulates both levels of phospho-eIF2α and the number of VA-induced SGs (Figure 4G and 4F, respectively). Although PERK is simultaneously activated by ER and oxidative stresses [41]; the ROS scavenger NAC indicates that oxidative stress does not contribute significantly to VA-induced SG assembly nor to the phosphorylation of eIF2α, unlike SA (Figure 4D and 4E). Finally, eIF2α phosphorylation is absolutely required for VA-induced SG assembly, as VRB fails to promote SG assembly in S51A MEFs (Figure 4C). Such dependence of VA-induced SG formation on eIF2α phosphorylation is in contrast to the reported effects of sodium selenite, another chemotherapy drug that affects both eIF2α phosphorylation and mTOR ([13], discussed below).

In addition to PERK, VAs also modulate the activity of mTOR (Figure 5), a kinase implicated in may aspects of cellular metabolism [42, 43]. VAs stimulate dephosphorylation of 4E-BP1 and thereby promote its association with m^7^GTP-bound eIF4E, thus causing concomitant displacement of eIF4G/eIF4A from cap-bound eIF4E (Figure 5A and 5B). Although VRB-induced down-regulation of mTOR is evident from the 4E-BP1 dephosphorylation, VRB does not impact the mTOR/p70S6K/ph-RPS6 axis, and VRB-induced SG assembly does not require RPS6 phosphorylation (Figure 5D). In contrast, depletion of 4E-BP1 inhibits VRB-induced SG assembly (Figure 5E). This data is in agreement with the role of 4E-BP1 in promoting selenite-induced SGs [13]. In addition, the cooperative involvement of mTOR and eIF2α kinases in VA-induced SG formation is also consistent with a report from Lykke-Andersen laboratory [44], indicating that mTOR cooperates with GCN2 kinase to regulate translation of with 5′-terminal oligopyrimidine tracts (5′TOP) mRNAs [44, 45] under conditions of nutrient starvation and stress.

However, there are significant differences in molecular mechanisms between VAs and selenite on SG formation and cell survival. First, selenite significantly decreases levels of eIF4G expression [13] thus affecting levels of translationally competent eIF4F complexes, while VAs do not affect levels of eIF4G. Second, selenite causes robust dephosphorylation of ph-RPS6 while VA drugs do not. Third, selenite-induced SGs are compositionally different from VA-induced. In contrast to VA-induced SGs, they lack canonical SG marker eIF3, demonstrate reduced recruitment of small ribosomal proteins and lack signaling molecules within them (such as RACK1) [13]. Finally, selenite-induced SGs are pro-apoptotic while VA-induced are pro-survival [13].

Chemotherapy drugs induce SGs that are either pro-apoptotic (e.g. selenite [13]) or anti-apoptotic (e.g. Sorafenib [34]). SG-mediated effects on cell survival do not appear solely mediated by the down-regulate of global cellular translation and selectively up-regulated translation of mRNAs encoding pro-survival, stress response and anti-apoptotic proteins. SGs also recruit specific signaling molecules [5]. VRB-induced SGs contain canonical core components including poly(A)-mRNAs, 40S ribosomal subunits, and translation initiation factors shared by SA-induced SGs. However, VRB-induced SGs contain lower amounts of the signaling proteins Rsk2, TRAF2 and RACK1 than SA-SGs (Figure 2). The consequences of the association of signaling molecules with SGs are complex, and their effects on cell metabolism and viability depend on the nature and duration of stress. For example, RACK1 may be both pro-apoptotic and anti-apoptotic. In one report, sequestration of RACK1 into SGs has a negative impact on the stress-activated p38 and JNK (c-Jun N-terminal kinase)/MAPK pathways leading to inhibition of apoptosis [11]. In another report, RACK1 appears to exert pro-apoptotic effects by interacting with apoptosis-related proteins such as BAX, a member of BCL-2 family [46].

As we cannot predict whether reduced association of these molecules with SGs is beneficial for cell resistance to VRB, we directly quantified the effect of VRB-induced SGs on cell survival. First, depletion of PERK, 4E-BP1 or both, significantly decreases viability of cells in the presence of VRB (Figure 6A-6B). Second, PERK- and 4E-BP1-depleted cells initiate apoptosis at lower concentrations of VRB relative to control cells (Caspase 3 cleavage under 60 and 70 μM, Figure 6B), in agreement with immunofluorescence data quantifying apoptotic cells (Figure 6C). Third, the viability of VRB-treated cells directly correlates SG formation (Figure 6D-6E). We employed a panel of genetically modified U2OS cell lines that either allow or prohibit SGs formation in response to various stresses [30]. U2OS cells deleted for both SG nucleators G3BP1 and G3BP2 (ΔΔG3BP1/2 cells) do not form SGs in response to VRB treatment in contrast to WT U2OS or ΔΔG3BP1/2 cells reconstituted with G3BP1 (Figure 6D). Similarly, ΔΔG3BP1/2 cells reconstituted with the site-specific G3BP1 mutant S149A (SG-competent) but not with G3BP1 S149E (SG-incompetent) assemble VRB-induced SGs (Figure 6D). The SG-competence of these cells parallels their ability to resist cell death over a range of VRB concentrations (60-100 μM, Figure 6E-6F). In summary, PERK/4E-BP1-induced translation inhibition and SG formation promote cell survival in response to VRB treatment.

What are the functional and clinical implications of our studies? As suggested, SGs are critical for cellular adaptation to diverse stresses. While SGs are classically induced by stresses that are environmental (such as heat shock or hypoxia) or physiological (viral infections), chemotherapy also constitutes an environmental factor that induces SGs. The key point is that cells use the same adaptive responses to both physiological and external factor-induced stimuli in order to survive. This represents both a problem and a unique clinical opportunity to target cancer cells for elimination. On one hand, some chemotherapy drugs induce SG formation which promotes survival and counteracts the toxic effects of the drugs. On the other hand, it may be possible to use combination therapies to damage cancer cells using chemotherapy (e.g. microtubule network disruption) while concurrently disabling SG formation. Clearly, further studies are required to determine how SGs regulate cell survival and affect chemotherapy treatment.

## MATERIALS AND METHODS

### Cell culture

Human osteosarcoma cells (U2OS), breast adenocarcinoma cells (MCF-7), lung adenocarcinoma epithelial cell (A549), cervical carcinoma cells (SiHa), mouse embryonic fibroblasts (MEFs) with/without S51A mutation of eIF2α, and ph-RPS6^−/−^ MEFs (gift of Dr. Oded Meyuhas, The Hebrew University of Jerusalem, Israel) were grown in Dulbecco's Modified Eagle Medium, DMEM (Sigma-Aldrich) with 10% fetal bovine serum (Sigma-Aldrich) and Penicillin-Streptomycin cocktail (Sigma-Aldrich).

### Antibodies and anticancer drugs

The following antibodies have been used in this study. Anti-G3BP (cat. sc-81940; 1:200 dilution for IF), anti-eIF4G (sc-11373; 1:200 dilution for IF, 1:1000 for WB), anti-eIF3b (sc-16377; 1:200 dilution for IF), anti-eIF4E (sc-9976; 1:200 dilution for IF, 1:1000 for WB), anti-FXR1 (sc-10554, 1:200 dilution for IF), anti-betaTubulin (sc-47751, 1:200 dilution for IF), and anti-Rack1 (sc-17754; 1:1000 dilution for WB) were purchased from Santa Cruz Biotechnology, Inc. Anti-YB-1 (cat. #4202; 1:200 dilution for IF), anti-eIF4A (#2490, 1:1000 dilution for WB), anti-total-4E-BP1 (#9452; 1:1000 dilution for WB) anti-total-eIF2α (#2103, 1:1000 dilution for WB), anti-P-rpS6 (#2211; 1:1000 dilution for WB), anti-nonP-4E-BP1 (#4923, 1:1000 dilution for WB), P-4E-BP1 in Thr37/46 (#2855, 1:1000 dilution for WB), P-4E-BP1 in Ser65 (#9451, 1:1000 dilution for WB), PRAS40 (#2691, 1:1000 dilution for WB), Caspase 3 total (#9662, 1:1000 dilution for WB) and Cleaved Caspase 3 (#9664, 1:400 dilution for IF) were purchased from Cell Signaling Technology. Anti-Stau1 (cat. 14225-1-AP; 1:500 dilution for IF) and PERK (20582-1-AP, 1:1000 dilution for WB) were purchased from ProteinTech. Anti-P-eIF2α (cat. Ab32157; 1:1000 dilution for WB) was purchased from Abcam. The secondary antibodies for WB, i.e. Peroxidase AffiniPure Donkey Anti-Mouse IgG (cat. 715-035-150) and Peroxidase AffiniPure Donkey Anti-Rabbit IgG (711-035-152) were purchased from Jackson ImmunoResearch. The secondary antibodies for IF included Cy™2 AffiniPure Donkey Anti-Mouse IgG (cat. 715-225-150), Cy™3 AffiniPure Donkey Anti-Rabbit IgG (711-165-152) and Alexa Fluor^®^ 647 AffiniPure Bovine Anti-Goat IgG (805-605-180) and were purchased from Jackson ImmunoResearch.

Vinorelbine ditartrate (VRB) was purchased from TSZ CHEM and Vinorelbine tartrate, as the commercially available anticancer drug Navelbine (VRB*), were purchased from Pierre Fabre Médicament. Vinblastine (VBL) and Vincristine (VCR) were purchased from BioTang Inc. Paclitaxel (PCX), Etoposide, Cycloheximide, Puromycin, Thapsigargin and Sodium Arsenite (NaAsO_2_) were purchased from Sigma-Aldrich.

### Immunofluorescence microscopy

SG immunofluorescence was done as described [10, 47]. Cells were fixed in 4% paraformaldehyde and permeabilized in 100% cold methanol (−20°C). Then, samples were incubated with blocking buffer (5% Horse Serum in PBS) for 1h. Cells were incubated with primary and secondary antibodies for at least 1h each and washed twice with PBS in between incubations. Hoechst 33258 was used together with the secondary antibodies in order to stain the nuclei. Cover slips with cells were mounted in polyvinyl mounting medium. Cells were imaged using an Eclipse E800 Nikon or AxioImager Carl Zeiss microscopes and photographed with either a SPOT CCD or a Pursuit CCD camera (both from Diagnostic Instruments) using the manufacturer's software. The images were analyzed and merged using Adobe Photoshop CS3.

### Fluorescence *in vitro* hybridization (FISH)

10^5^ cells grown on coverslips were fixed in 4% formaldehyde in PBS (15 min) and subsequently permeabilized in 96% cold methanol (15 min). PerfectHyb^™^ Plus Hybridization Buffer (Sigma-Aldrich, H7033) was used to block samples (30 min at 42°C) and hybridize the probe (synthetic oligo-dT_40_ labeled with cy3 or cy5) for 2h at 42°C. Then, samples were washed three times with 2xSSC (the first time with pre-wormed and subsequent times with room temperature buffer) and one time with PBS. 0.5 mg/ml UltraPure™ BSA (Ambion, AM2616) was used to block cells and apply primary and secondary antibodies (including Hoechst 33258). Finally, coverslips with cells were washed twice with PBS and mounted in polyvinyl mounting medium.

### Quantification of stress granules

The percentage of stress granules in a cell population was quantified by manual counting of approximately 700 cells with/without stress granules using Adobe Photoshop. Quantification of band intensity in WB technique was done using ImageJ software.

### Immunoblotting

Cells were grown in 6-well plates until 80% confluence. They were washed with HBSS buffer and solubilized in the lysis buffer (5mM MES, pH 6.2, and 2% SDS). 10s microwave heating was applied followed by 2 × 2 min sonication at 4°C. Lysates were denatured in boiling water and cooled down. Proteins were precipitated in 60% acetone at −20°C overnight. Lysates were then centrifuged (13.500 rpm, 4°C, 15 min) and supernatant was carefully removed and discarded. Pellets were dissolved in 1x Laemmli loading buffer, proteins were separated in 4-20% SDS-PAGE gels (BioRad) and transferred to nitrocellulose membranes using Trans-Blot^®^ Turbo™ system (BioRad). After 1h blocking in 2% milk in TBS-Tween, membranes were incubated with primary and secondary antibodies for a minimum 1h (membranes were also washed 5x after each type of antibodies). Finally, HRP-conjugated secondary antibodies were detected with SuperSignal West Pico Chemiluminescent Substrate (ThermoScientific) according to the manufacturer instruction.

### 7-Methyl GTP sepharose chromatography

The method was performed as previously described [48]. U2OS cells were grown to 80% confluence in 6-well plates under standard conditions. Cells were scraping into lysis buffer (50 mM Tris-HCl, pH 7.2, 100 mM NaCl, 1mM EDTA, 0.5% NP-40) supplemented with protease and phosphatase inhibitors (Thermo Scientific). Lysates were tumbled at 4°C for 15 min. Cells debris and nuclei were removed by centrifugation in microfuge tubes (20 min, 13,200 rpm, 4°C), and the cytoplasmic fraction was incubated with immobilized m^7^GTP-Sepharose (Jena Biosciences) for 1 h at 4°C with tumbling. Approximately 200 μl of m^7^GTP-Sepharose was used per sample. After incubation, beads were washed three times with lysis buffer, and m^7^GTP-bound protein complexes were collected on Micro Bio-Spin Chromatography Columns (Bio-Rad). The proteins were eluted from the column with 1x SDS-PAGE loading buffer and analyzed by western blotting using protein-specific antibodies (eIF4G, eIF4A1, eIF4E, 4E-BP1).

### siRNA-mediated knock-down assay

10^5^ U2OS cells were seeded in the 6-well plates and grown for 24 h. Then, the first transfection was done with the following substrates: 100 pmol siRNA (Thermo Scientific, Dharmacon, all are SmartPools), 2.5 μl Lipofectamine 2000 (Invitrogen) in OPTI-MEM (Life Technologies) and cells were incubated with siRNA for 24 h. Between the first and second transfection cells were cultured in DMEM with 10% fetal bovine serum for 24 h. The second transfection was done with the same conditions as the first. Finally, cells were collected and counted.

### Apoptosis assays

Cells were treated with VRB for 24h in 6-well plates (for western blot analysis of total/cleaved Caspase 3, 2×10^5^ cells) or on cover slips (for immunofluorescence with detection of cleaved Caspase 3, 10^5^ cells). The total/cleaved detection of Caspase 3 in western blot was done using Caspase 3 total (Cell Signaling, #9662) and cleaved Caspase 3 fragment in immunofluorescence was done using Cleaved Caspase 3 (Cell Signaling, #9664).

### Cytotoxicity/viability assay

CytoTox-Glo™ Cytotoxicity Assay was done according to manufacturer instruction (Promega), in 96-well plate format (10^3^ cells/well). Briefly, 100 μl of AAF-Glo™ Reagent was added to each well and the plate was incubated at room temperature for 15 min. The luminescence (determination of living cells) was measured and 100 μl of Lysis Reagent with digitonin was added and incubated for 15 min. Again, the luminescence was measured (determination of total cells). Viability of cells was measured as percentage of dead cells.

### Polysomes profiles

Cells were washed with cold HBSS, scrape-harvested directly into lysis buffer (10 mM HEPES pH 7.5, 125 mM KCl, 5 mM MgCl_2_, 1 mM DTT, 100 μg/mL cycloheximide, 100 μg/mL heparin, 1% NP40 made in DEPC-treated water), supplemented with RNasin Plus inhibitor (Promega) and HALT phosphatase and protease inhibitors (Thermo Scientific). Lysates were rotated at 4^°^C for 15 min, cleared by centrifugation for 10 min at 12,000 *g*, and supernatants loaded on pre-formed 17.5-50% sucrose gradients made in gradient buffer (10 mM HEPES pH 7.5, 125 mM KCl, 5 mM MgCl_2_, 1 mM DTT). Samples were centrifuged in a Beckman SW140 Ti rotor for 2.5h at 35,000 rpm, then eluted using a Brandel bottom-piercing apparatus connected to an ISCO UV monitor, which measured the eluate at OD 254.

## SUPPLEMENTARY MATERIAL FIGURES


