# Supplementary material for: Vinca alkaloid drugs promote stress-induced translational repression and stress granule formation
Source: Oncotarget. 2016 Apr 13;7(21):30307–22. doi: 10.18632/oncotarget.8728 (PMC5058682; doi:10.18632/oncotarget.8728)
Supplement: Supplementary file 1 [file oncotarget-07-30307-s001.pdf]

Vinca alkaloid drugs promote stress-induced translational repression and stress granule formation

Supplementary Material

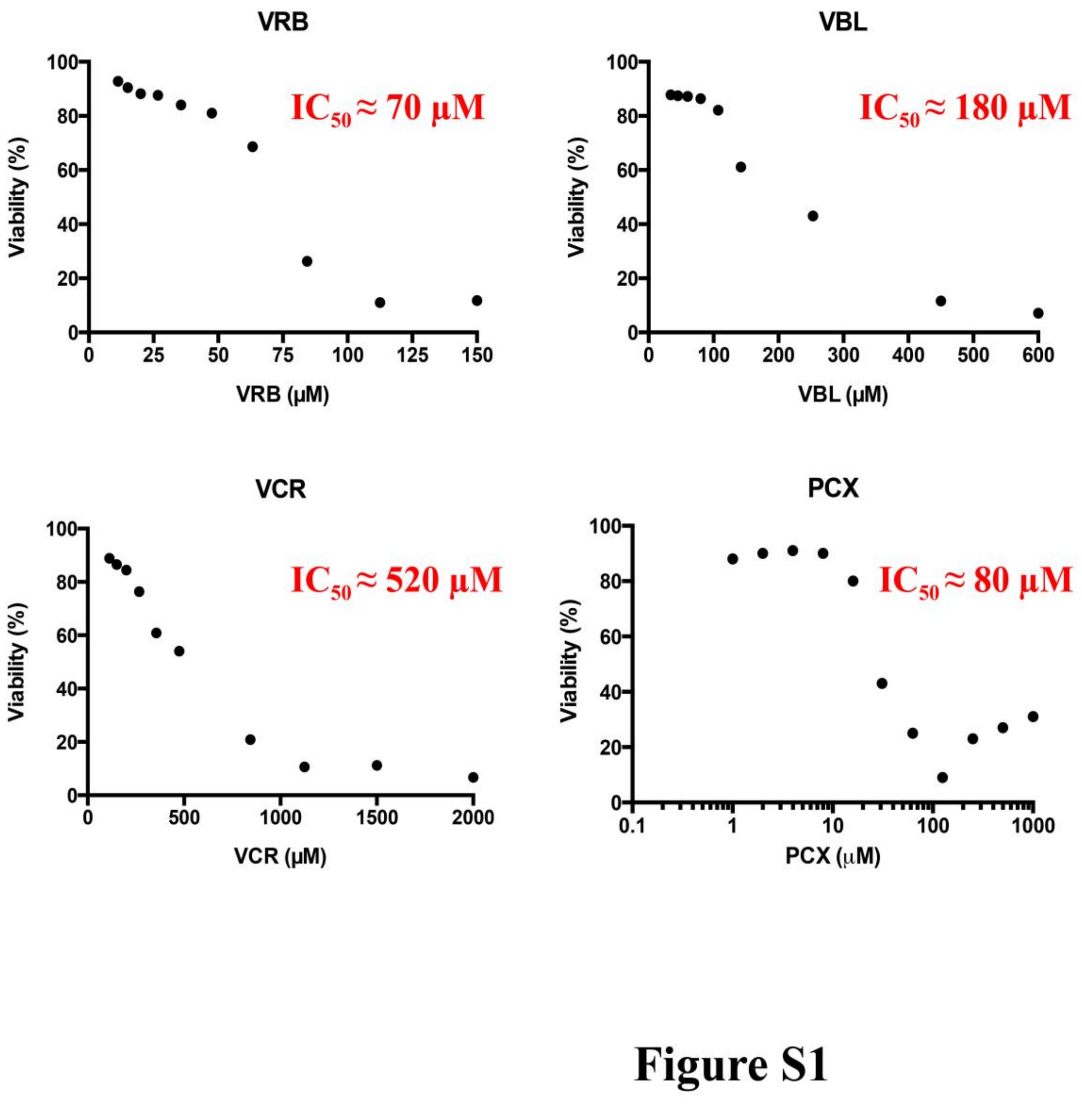

Supplementary Figure 1. Determination of the half maximal inhibitory concentrations (IC50) of VAs and PCX in U2OS cells.

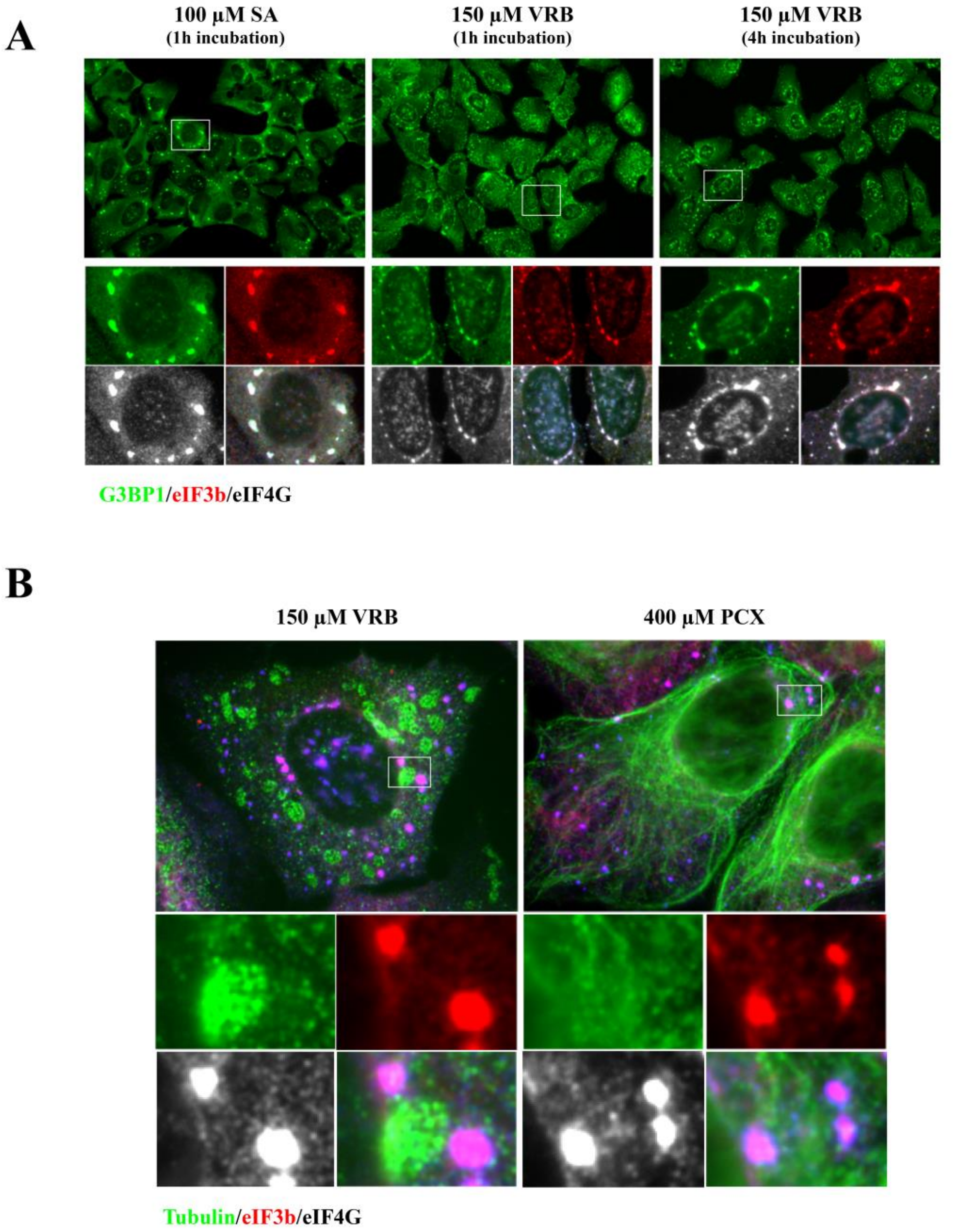

Figure S2

Supplementary Figure 2. VRB promotes SG formation in U2OS cells. Cells were treated with SA (100 µM) for 1 hour or VRB (150 µM) for 1 or 4 hours. SG were visualized by staining with G3BP1 (green), eIF3b (red) and eIF4G (blue, shown as gray) antibodies. **B**: VRB disrupts and PCX stabilizes microtubule networks. U2OS were treated with 150 µM VRB or 400 µM PCX for 1 hour. Cells were stained for the microtubule marker β-Tubulin (green), SG markers eIF3b (red) and eIF4G (blue, shown as gray).

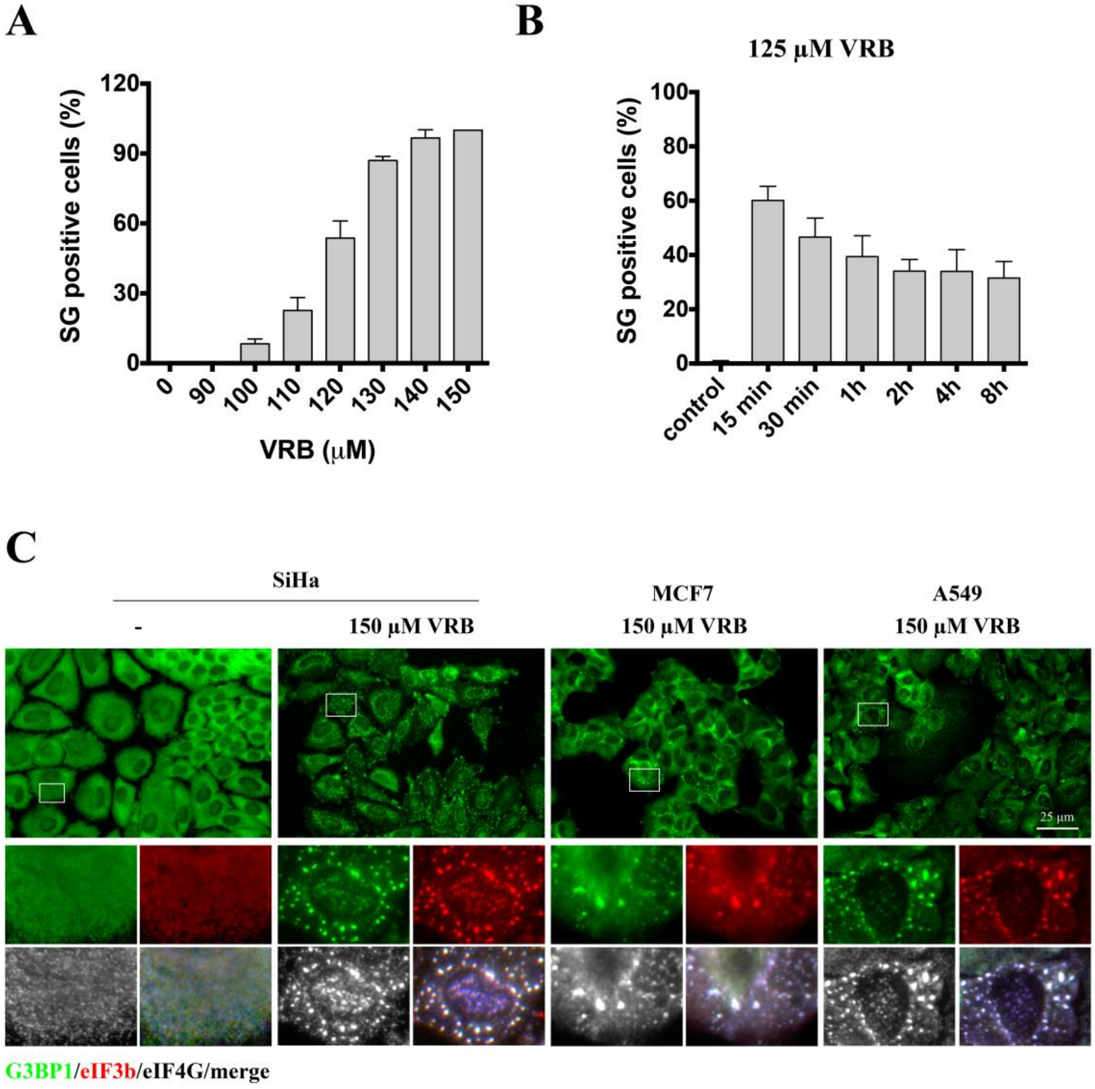

Figure S3

Supplementary Figure 3. VRB promotes SG formation in various cells. **A**: Percentage of SG-positive U2OS cells under various concentrations of VRB (0-150 μM) was determined by quantifications of G3BP1-positive SGs. **B**: VRB induces rapid SG formation in U2OS cells. **C**: VRB-induced SG formation in various cancer cell lines. Cervical (SiHa), breast (MCF7) and lung (A549) cancer cell lines were treated with 150 μM of VRB. Untreated SiHa cells also shown (-) as a control. SGs were visualized by staining with markers G3BP1 (green), eIF3b (red) and eIF4G (blue, shown as gray).

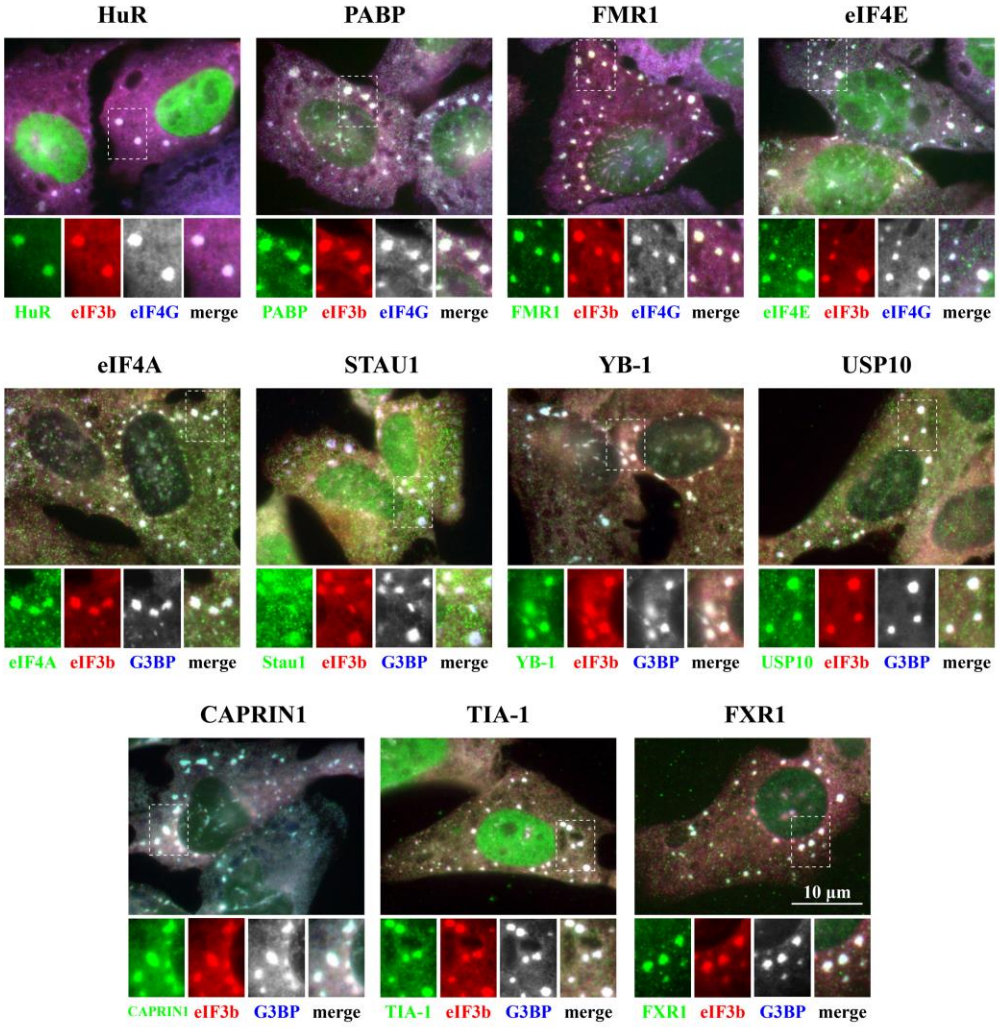

Figure S4

Supplementary Figure 4. Composition of VRB-induced SGs. Composition of VRB-induced SGs was determined by co-localization of SG-associated proteins HuR, PABP, FMR1, eIF4E, eIF4A, STAU1, YB-1, USP10, CAPRIN1, TIA-1 and FXR1 (all in green) with canonical SG markers eIF3b (red), eIF4G or G3BP1(blue, shown as gray) .

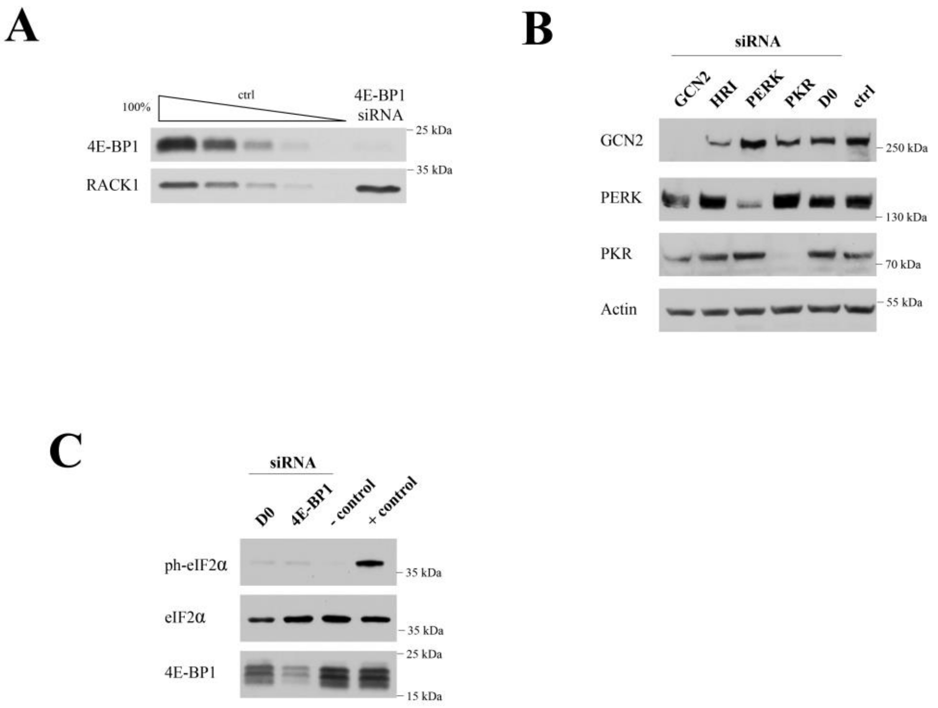

Figure S5

Supplementary Figure 5. Efficiency of knock down of 4E-BP1 and eIF2α kinases. **A-B**: Whole cell lysates from U2OS cells treated with control siRNA (ctrl) and 4E-BP1 siRNA (**A**) or two control siRNAs (Do and Ctrl) and eIF2α -specific kinases (PERK, PKR, GCN2 and HRI) (**B**) were processed for Western Blotting using 4E-BP1 (**A**) or PERK-, PKR-, GCN2 or HRI- (**B**) specific antibodies. Dilution series of lysates from control siRNA-treated cells (100%, 50%, 25% and 12.5%) are used (triangle) to determine the relative efficiency of 4E-BP1 depletion (**A**). RACK1 serves as loading control (**A**). Actin serves as loading control for eIF2α kinases depletion (**B**). **C**: Evidence that 4E-BP1 depletion does not affect eIF2α phosphorylation. Depletion of 4E-BP1 was done as described in S5A. siRNA treatment with control siRNA (D0) was used as control for non-specific effects. No treatment (- Control) or treatment with sodium arsenite (+Control) was used for the detection of eIF2α phosphorylation levels. Non-phosphorylated form of eIF2α served as a loading and reference controls.
